# Novel Insights Into Molecular Mechanism of Mitochondria in Diabetic Cardiomyopathy

**DOI:** 10.3389/fphys.2020.609157

**Published:** 2021-01-18

**Authors:** Jing Bai, Chuanbin Liu, Pingjun Zhu, Yang Li

**Affiliations:** ^1^Medical School of Chinese PLA, Beijing, China; ^2^Department of Cardiology, The Sixth Medical Center, Chinese PLA General Hospital, Beijing, China

**Keywords:** diabetic cardiomyopathy, mitochondria, mitochondrial morphology, energy metabolism, oxidative stress, calcium homeostasis, mitochondrial autophagy

## Abstract

Cardiovascular complication is one of the significant causes of death in diabetic mellitus (DM) in which diabetic cardiomyopathy, independent of hypertension, cardiac valvular disease, and coronary atherosclerosis, occupies an important position. Although the detailed pathogenesis of diabetic cardiomyopathy remains unclear currently, mitochondrial morphological abnormality and dysfunction were observed in diabetic cardiomyopathy animal models according to much research, suggesting that mitochondrial structural and functional impairment played an integral role in the formation of diabetic cardiomyopathy. Thus, we have summarized the effect of mitochondria on the process of diabetic cardiomyopathy, including abnormal mitochondrial morphology, mitochondrial energy metabolism disorder, enhanced mitochondrial oxidative stress, mitochondrial unbalanced calcium homeostasis, and mitochondrial autophagy. Based on the above mechanisms and the related evidence, more therapeutic strategies targeting mitochondria in diabetic cardiomyopathy have been and will be proposed to delay the progression of the disease.

## Introduction

The prevalence rate of diabetic mellitus (DM) shows such a significant increase that it has long been an epidemic disease that badly affects human health. About half a billion people are living with diabetes worldwide and the number is estimated to increase by 25% in 2030 and 51% in 2045 (Saeedi et al., 2019; Cao et al., 2020). The early cardiac manifestation of DM is diastolic dysfunction with or without systolic reserve dysfunction while systolic dysfunction, and even congestive heart failure, may appear in a later stage (Marwick et al., 2018). Although the underlying disease of diabetic cardiomyopathy (DCM) is DM, it is a kind of primary injury that is independent of hypertension, cardiac valvular disease, and coronary atherosclerosis (Dillmann, 2019; Cuijpers et al., 2020).

Recent studies demonstrated that mitochondria may play an indispensable role in many links to the genesis of DCM. It was reported that the activity of mitochondrial respiratory chain-related enzymes in Zucker diabetic obese rats decreased significantly (Raza et al., 2012; Daiber and Münzel, 2020), and similar mitochondrial respiratory dysfunction was observed in Type 2 diabetic mellitus (T2DM) mice model (Sloan et al., 2011; Pham et al., 2014). Montaigne et al. (2014) discovered fragmented mitochondria in cardiomyocytes of diabetic patients, and Anderson et al. (2009) directly confirmed mitochondrial respiratory dysfunction in the atrial myocytes of T2DM patients, providing strong evidence to link mitochondrial impairment with diabetes incidence (Heusch, 2019). In this study, we will summarize the effect of mitochondria on the process of DCM and the underlying mechanism. In addition, we briefly introduced the latest progress of DCM therapy targeting mitochondria.

## Molecular Mechanisms of Mitochondria in Diabetic Cardiomyopathy

### Abnormal Mitochondrial Morphology

In normal conditions, mitochondrial fusion and fission keep in balance dynamically, which is important to maintain the normal physiological function of mitochondria and cells (Lanna and Dustin, 2016; Larson-Casey et al., 2020). Once the balance is disrupted, the result is markedly reduced energy synthesis and increased reactive oxygen species (ROS) production, thus promoting cell death and disease progression. In the literatures, the molecules related to mitochondrial fusion are Mitochondrial Fusion Protein (Mfn1 and Mfn2), Optic Atrophy Factor (OPA1), while the main factors mediating fission are Dynamin-Like Protein 1 (DLP1, also referred to as DRP1), Mitochondrial Fission Protein (Fis1), and Mitochondrial Fission Factor (Mff). Makino et al. (2010) reported that the reduction of OPA1 was the trigger event of mitochondrial fission, and chronic hyperglycemia might inhibit the expression of fusion protein OPA1 (Klinge, 2020). After suppressing mitochondrial fission, the production of ROS was reduced (Molina et al., 2009). Moreover, the abnormal expression of fusion protein and fission protein led to cardiac defect both in structure and function, and damaged mitochondrial fusion in the mature heart tissue, thereby disrupting heart homeostasis (Galloway and Yoon, 2015; Zhou et al., 2020). The research of Parra et al. (2014) demonstrated that insulin was related to mitochondrial dynamics, especially mitochondrial fusion, and the defect in insulin signal of diabetic patients contributed to an impaired expression of Mitochondrial Fusion Protein Mfn1, Mfn2, and OPA1. Cardiomyocytes in DCM patients manifested impaired systolic function, augmented oxidative stress, diminished energy production, fragmented mitochondria, and low expression of Mfn1, which was in negative correlation to HbA_1_c level (Montaigne et al., 2014). DRP1 overexpression caused mitochondrial dysfunction and insulin resistance in cardiomyocytes, which could be relieved after DRP1 silence (Watanabe et al., 2014; Kuczynski and Reynolds, 2020). Lipid overload increased A-kinase anchor protein 121 ubiquitination, regulated DRP1 phosphorylation, and altered OPA1 processing (Tsushima et al., 2018; Ding et al., 2020). OPA1 mutations led to abnormal mitochondrial morphology and increased ROS production, as well as susceptibility to oxidative stressors (Tang et al., 2009). Researchers indicated that DRP1 overexpression or Mfn1 suppression markedly raised ROS production (Huang et al., 2016). Genetic fusion interventions (inducing mitochondrial elongation) were associated with decreased mitochondrial ROS production, while fission interventions (resulting in mitochondrial fragmentation) stimulated mitochondrial ROS production (Picard et al., 2013; Jusic and Devaux, 2020). Therefore, there is an inextricable link between mitochondrial morphology and DCM: Altered mitochondrial morphology is not only causal for but also consequential to DCM, hence intensifying oxidative damage through reciprocal amplification, which is important to the process of DCM. The latest research of Hu et al. (2019) found that in T2DM db/db hearts, mitochondrial fission was exceedingly vigorous, and the lessened Mfn2 might be due to reduced expression of peroxisome proliferator-activated receptor α (PPARα) and binding of PPARα to Mfn2 promoter. In the view of the fact that mitochondrial dynamics is actually the basis of mitochondrial function, more profound research is needed to effectively intervene mitochondrial fusion and fission in DM in order to delay the progression to DCM.

### Mitochondrial Energy Metabolism Disorder

Cardiomyocytes are high energy consuming cells whose energy production mainly occurs in mitochondria. Under physiological conditions, the fatty acid β-oxidation constitutes about 70% of the source of energy in heart, with the remaining part produced from the oxidation of other nutrients, such as glucose, ketone bodies, lactate, and amino acid (Bertero and Maack, 2018; Ma et al., 2020). It is worth noting that compared with glucose, fatty acid as energy metabolic substrates requires about 12% more oxygen to produce the same amount of ATP. However, the fatty acid β-oxidation in diabetic heart increases while the glucose oxidation decreases, aggravating hypoxia in myocardium with microangiopathy. In DCM patients, ATP is mainly synthesized by mitochondrial fatty acid β-oxidation, which can lead to increased oxygen consumption and respiratory dysfunction in mitochondria.

In addition, there is a kind of uncoupling protein (UCP) with the function of ion channel on the mitochondrial inner membrane, which induces the decrease of ATP production by consuming the proton power of the mitochondrial membrane (Demine et al., 2019). UCP is easily activated by ROS, norepinephrine, and fatty acid. It can make the protons pumped out from the process of electron transfer in the mitochondrial respiratory chain re-enter the mitochondrial matrix through the proton channel formed by UCP. This kind of “proton leakage” releases the electrochemical potential energy of protons in the form of heat, and the oxidative phosphorylation appears “uncoupling” because it is not coupled with ATP synthase. Five UCP subtypes have been found in mammals, and UCP2 and UCP3 occupy the dominant position in myocardial mitochondria. It was revealed that mitochondrial uncoupling in cardiomyocytes of db/db mice was enhanced, and the function of mitochondrial respiratory chain was impaired. It was also reported that the expression of UCP3 increased after cardiac ischemia in db/db mice, with damaged mitochondrial and impaired cardiac energy efficiency (Banke and Lewandowski, 2015; Takov et al., 2020), which was confirmed in the hearts of rats fed with high-fat diet. However, there are controversies. Some scholars were convinced that UCPs can protect against free radical damage by regulating mitochondrial respiration, inducing reduced production of ROS (Dludla et al., 2018).

In diabetes or insulin resistance, increased myocardial fatty acid content in patients with DCM can cause the activation of PPARα, which facilitates the inhibition of pyruvate dehydrogenase kinase and the impairment of glucose oxidation ability, thereby increasing mitochondrial fatty acid uptake and subsequently causing energy consumption (Mirza et al., 2019). In addition, excessive fatty acid accumulation is also considered to be directly related to diabetic myocardial toxic injury and dysfunction, which is mainly caused by lipid intermediate metabolites, such as ceramide, diacylglycerol, long chain phosphatidyl coenzyme A, and so on (Chong et al., 2017). Overall, the metabolism of fatty acid by mitochondria increases the oxygen consumption of heart, resulting in changes in the structure and function of heart, thereby inducing DCM.

### Enhanced Mitochondrial Oxidative Stress

In the physiological state, only a very small amount of oxygen is reduced to ROS by single electron reduction (Ghaemi Kerahrodi and Michal, 2020; Scialò et al., 2020). However, in diabetes, due to changes, such as high glucose, high lipid, insulin resistance, calcium signal disorder, and enhanced mitochondrial uncoupling, more nicotinamide adenine dinucleotide (NADH) and flavin adenine dinucleotide (FAD) would flow to mitochondrial respiratory chain, causing hyperpolarization of the mitochondrial inner membrane, suppression of electron transfer in complex III, and excessive generation of ROS (Shah and Brownlee, 2016; Zhang et al., 2020). Thus, the antioxidant capacity of the body is relatively insufficient, bringing about the enrichment of a large amount of ROS, which enhances the damage of oxidative stress on proteins, nucleic acids, and lipids. Finally, the destruction of the structural, physiological, and metabolic mechanisms is induced, as well as abnormal regulation in cells and tissues.

In cardiac mitochondria of type 2 diabetic mice, an increase of superoxide free radicals was found, and inhibition of mitochondrial oxidative stress could delay the occurrence of DCM in streptozotocin (STZ)-induced diabetic mice. Anderson et al. (2009) confirmed that the oxidative stress of atrial mitochondria was enhanced and the mitochondrial function was impaired in DM patients (Anderson et al., 2009). Sun et al. (2014) studied diabetic mice *in vivo* and *in vitro*, illustrating that the morphology of mitochondria in cardiomyocytes changed and the level of ROS was elevated in hyperglycemic mice. The above studies provide some evidence for the involvement of mitochondrial ROS in the pathogenesis of DCM. Besides, ROS can damage the structure of diabetic myocardial mitochondria and further damage the function of mitochondria by facilitating the opening of mitochondrial permeability transition pore (mPTP) on mitochondrial inner membrane. Normally, mitochondria produce an electrochemical gradient across the membrane by electron transfer, and ATP is generated by ATP synthase (Tsuyama et al., 2017; Vlacil et al., 2020). Following the overload of ROS in diabetes, mPTPs, which are very sensitive to ROS, turn to be opened, leading to membrane potential depolarization, reversed transport of ATP synthase, exhaustion of cell energy, and cardiomyocyte death. ROS can also make mPTPs sensitive to calcium ions, resulting in calcium overload and further aggravating membrane permeability. In addition to mitochondrial ROS, it has also been reported that there is an increase of NADH phosphate (NADPH) oxidase-derived ROS in the myocardium of ob/ob mice, STZ mice, and Zucker fa/fa rats, and it has been discovered that direct or indirect activation of antioxidant enzymes can effectively prevent protein nitrification and inflammation, and can reverse DCM damage, suggesting that two pathways of increasing ROS exist in diabetic myocardium, i.e., a mitochondrial way and an extramitochondrial way (Cong et al., 2015). Notably, mitochondria are not only the major sites of ROS production, but also the main targets of ROS attacks. In comparison with other intracellular structures, ROS is more likely to damage mitochondrial membrane, mitochondrial DNA (mtDNA), and its encoded proteins (Vaeyens et al., 2020). To sum up, reducing oxidative stress of myocardial mitochondria or improving the antioxidant capacity of cells is expected to improve DCM.

### Mitochondrial Unbalanced Calcium Homeostasis

Normal cardiac function is closely related to the maintenance of intracellular Ca^2+^ homeostasis, which regulates metabolism, muscle contraction, and signal transduction (Jia et al., 2018). In the excitation contraction coupling of myocardium, Ca^2+^ gets into the cytoplasm *via* voltage sensitive L-type calcium channels after sarcolemma depolarization, triggering the release of Ca^2+^ from the sarcoplasmic reticulum. During the diastolic process, Ca^2+^ is transferred back to sarcoplasmic reticulum, followed by the surplus Ca^2+^ being pumped out through sarcolemma Na^+^/Ca^2+^ exchanger and Ca^2+^ pump on the plasma membrane (Kanaporis and Blatter, 2017). Nevertheless, in DCM disrupted calcium homeostasis induced by the above transporters makes action potential duration increased and diastolic relaxation time prolonged (Jia et al., 2016).

In addition to the Ca^2+^ regulation of endoplasmic reticulum (ER), the role of mitochondrial Ca^2+^ regulatory disorders in DCM has attracted much attention in recent years. Mitochondria have the function of regulating Ca^2+^, storing Ca^2+^, and producing energy (Drago and Davis, 2016; Yu et al., 2016), and some laboratories have reported that there are mitochondrial Ca^2+^ regulatory disorders in the heart of diabetic animal models. It was demonstrated that Ca^2+^ overload in cardiomyocytes of diabetic patients might lead to respiration and oxidative phosphorylation damage, and increase of ROS. Ca^2+^, enters mitochondrial matrix mainly through mitochondrial calcium uniporter (MCU), which makes mitochondria acing in the buffering role to shape cytosolic Ca^2+^ signals (Baradaran et al., 2018). A very slight change in the concentration of Ca^2+^ in mitochondria can activate ATP synthase and promote the production of ATP [dissociation constant (*K*_d_) ≤ 2 nM; Kirichok et al., 2004; Kamer and Mootha, 2015]. Study indicated that in cardiomyocytes stimulated by high glucose, the expression of MCU and the concentration of Ca^2+^ in mitochondria decreased, accompanied by the disorder of glucose and lipid metabolism, and the above change also occurred in the heart of type 1 diabetic mice (Diaz-Juarez et al., 2016). In addition, many factors, such as accumulation of free fatty acid, increased oxidative stress, disordered Ca^2+^, decrease of mitochondrial membrane potential, and exhaustion of ATP in mitochondria etc., can cause the persistent high-level opening of mPTP (Zorov et al., 2014). This not only leads to the imbalance of intracellular Ca^2+^ regulation, but also promotes the release of many pro-apoptotic factors, which bind to apoptotic protease activator in order to induce caspase cascade reaction and promote cardiomyocyte death. Besides, mitochondrial Ca^2+^ overload and intracellular oxidative damage cause and affect each other, forming a vicious cycle, eventually leading to apoptosis or necrosis and influencing cardiac systolic and diastolic function (Joseph et al., 2016; Salin Raj et al., 2019). In my point of view, studies above suggest that impaired mitochondrial function in DCM cardiomyocytes can affect the regulation of Ca^2+^, but the exact molecular mechanism and signal pathway needs more profound investigation.

### Mitochondrial Autophagy

Mitochondrial autophagy (mitophagy) occurs under the stimulation of nutritional deficiency and cell senescence, when the depolarization of mitochondria appears and the damaged mitochondria are specifically wrapped into autophagosomes and then fused with lysosomes, thus completing the degradation of damaged mitochondria. With the in-depth study of autophagy, it was proved that mitophagy played protective roles. In Beclin1 or Atgl6 knocked mice, it was also observed that the expression of Pink and Parkin was increased, as well as the elevated level of manganese-containing superoxide dismutase, prompting that increased mitochondrial autophagy might improve myocardial harm in autophagy-deficient mice, which might be associated with the Ras related protein 9-dependent unconventional autophagy pathway (Xu et al., 2013; Smadja et al., 2020). Suppression of mitochondrial autophagy mediated by deacetylase Sirt3, the first member of the Sirtuin family located in the mitochondria of mammalian cells, can lead to diabetic myocardial damage (Wang and Zhou, 2020; Wang et al., 2020). Koncsos et al. (2016) reported that the expression of mitochondrial autophagy-associated protein BNIP3 decreased in prediabetic rats fed with high-fat diet, followed by myocardial diastolic dysfunction (Margadant, 2020). In a recent study, mitochondrial dysfunction and DCM were observed in diabetic mice, while the injection of Tat-Beclin1 reversed such DCM by activating mitochondrial autophagy, indicating mitophagy served as a critical quality control mechanism for mitochondria in heart during high-fat diet (Tong et al., 2019). Considering out previous studies, it can be speculated that in early stage of DCM, the decrease of autophagy causes the upregulation of mitophagy, which plays a positive role in myocardium. Then, the ability of mitochondrial clearance decreases, resulting in accumulation of impaired mitochondria and leading to myocardial damage.

## Potential Dcm Treatments Targeting Mitochondria

Mitochondria, as a therapeutic target in DM-related cardiovascular disease, have brought out widespread attention because of more comprehensive understanding of mitochondrial effects on DCM and the mechanisms of antidiabetic drugs (Gollmer et al., 2020). At present, a large quantity of antidiabetic drugs applied in clinical therapy have already directly or indirectly eased mitochondrial negative effects on DCM, such as metformin. Yang et al. (2019) demonstrated that metformin can activate AMPK pathway and improve autophagy through suppressing the mTOR pathway and relieving apoptosis in cardiomyocytes of neonatal mice with DCM. ER stress is considered a typical characteristic in DCM. A recent research showed that in mice without DM, activation of ER stress induced by thapsigargin damaged mitochondrial respiration, seemingly facilitating mPTP opening, and inducing mitochondrial oxidative stress (Chen et al., 2017). After being treated with metformin, these mice seemed to reverse their mitochondrial abnormalities in some degree, probably by activating protein kinase, indicating that metformin might be effective in the negative effects of mitochondria on DCM caused by ER stress as well as by other factors.

Recently, sodium glucose cotransporter 2 inhibitors (SGLT2i) have attracted much attention due to its function of improving cardiovascular outcomes in diabetic patients (Wiviott et al., 2019). Based on those clinical trial findings, the European Society of Cardiology has listed SGLT2i as a first-line therapy in diabetic patients with high or very high cardiovascular risk or existing cardiovascular disease (Cosentino et al., 2020). The myocardial mechanisms of SGLT2i have been suggested, in which mitochondria may play a pivotal role. The Na^+^/H^+^ exchanger 1 (NHE1) in heart has been confirmed as a target of SGLT2i. In primary cardiomyocytes of mice, treatment with empagliflozin, which is a representative drug of SGLT2i, restrains NHE1 flux and depresses cytosolic Ca^2+^ and Na^+^ levels, potentially by combining empagliflozin with NHE1 (Uthman et al., 2018; Ludwig et al., 2020). Baartscheer et al. (2017) reported that empagliflozin lowers cytosolic Ca^2+^ concentrations while raising mitochondrial Ca^2+^ concentrations. Accordingly, SGLT2i may alleviate the disruption of both cytosolic and mitochondrial Ca^2+^ homeostasis and may elevate ATP production by the activation of mitochondrial Ca^2+^-sensitive dehydrogenases. Besides, several drugs, such as MitoQ, MnTBAP, and MitoTempol, have been identified to attenuate mitochondrial defects targeting the reducing of mitochondrial oxidative stress (Ilkun et al., 2015; Escribano-Lopez et al., 2016). Fang et al. (2018) confirmed that resveratrol might alleviate cardiac oxidative stress, mitochondrial impairment, and myocardial fibrosis in diabetes induced by high glucose. Liu et al. (2018) explored the protection of spironolactone against DCM in STZ-induced diabetic rats and concluded that its cardioprotective effects were due to improving mitochondrial dysfunction and reducing cardiac fibrosis, oxidative stress, and inflammation (Liu et al., 2018). Hu et al. (2019) demonstrated that the reconstruction of Mfn2 restored mitochondrial membrane potential, inhibited mitochondrial oxidative stress, and ameliorated mitochondrial function in cardiomyocytes treated by high glucose and high-fat through facilitating mitochondrial fusion. Some drugs targeting mitochondrial energy metabolism, such as Pioglitazone, the agonist of PPAR-γ, have been the first-line treatments for DCM (Wassef et al., 2018). Unfortunately, most of the research is from preclinical study, suggesting that there is a long way to go in the treatment of DCM with mitochondria as a target.

## Discussion

**Figure 1:**
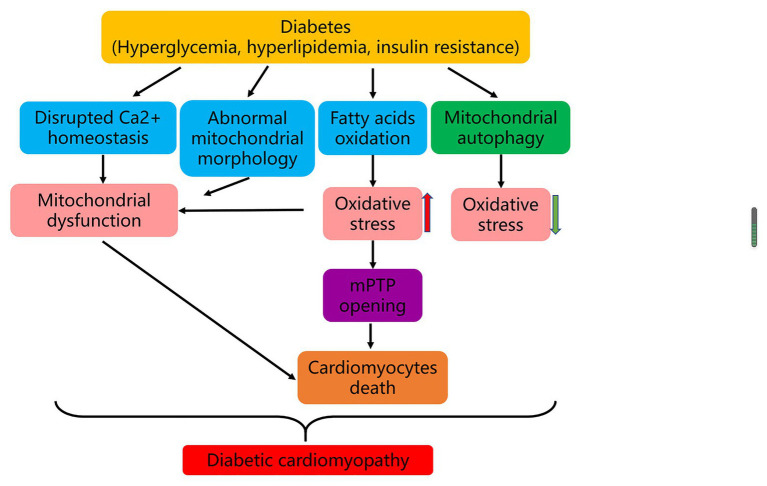
Mechanisms of DCM. In diabetes, hyperglycemia, hyperlipidemia, and insulin resistance result in disrupted Ca^2+^ homeostasis, abnormal mitochondrial morphology, and enhanced fatty acids oxidation. The latter changes further lead to mitochondrial dysfunction and strengthened oxidative stress, which induce cardiomyocytes death and eventually cause DCM. In the development of DCM, mitophagy may play a protective role.

In summary, mitochondrial impairment plays a critical role in the pathogenesis of DCM. Typically, 90% of intracellular ATP production is provided *via* mitochondrial oxidative phosphorylation. In T2DM, as the main source of ATP production in mitochondria, free fatty acid oxidation replaces part of glycolysis, with elevated mitochondrial ROS production and damaged oxidative phosphorylation. The change of mitochondrial Ca^2+^ treatment and the breakup of mitochondrial fission and fusion balance further exacerbate mitochondrial respiratory dysfunction and lead to cell death. Moreover, the mitochondrial dysfunction induced by enhanced oxidative stress also increases the opening of mPTP induced by Ca^2+^ overload, causing cardiomyocyte autophagy and myocardial necrosis. Notably, mitochondrial autophagy may play a protective role in the pathogenesis of DCM (Figure 1).

As such knowledge is mainly derived from animal models, it is essential that the effect of mitochondria on human DCM be further investigated in order to search for the potential treatment of DCM targeting mitochondria. In fact, some existing drugs, e.g., metformin, have the curative effect of reversing or at least relieving the mitochondrial negative influence on diabetic myocardium. Considering the universal mitochondria-related diseases in human beings, mitochondrial therapies used in practice will enable us to benefit from these new treatments.

## Author Contributions

JB, CL, PZ, and YL contributed to conception, drafted the manuscript, critically revised the manuscript, provided the final approval, and agreed to be accountable for all aspects of work ensuing integrity and accuracy. All authors contributed to the article and approved the submitted version.

### Conflict of Interest

The authors declare that the research was conducted in the absence of any commercial or financial relationships that could be construed as a potential conflict of interest.
